# Trends in AI-based diagnosis and intervention of metabolic diseases: a bibliometric analysis of the literature from 2000 to 2024

**DOI:** 10.3389/fmed.2025.1698366

**Published:** 2025-12-05

**Authors:** Biting Chen, Yinlu Wang, Xuhuan Xie, Boyan Fan, Wen Zhang, Guixiang Sun

**Affiliations:** 1 College of Traditional Chinese Medicine, Hunan University of Traditional Chinese Medicine, Changsha, China; 2 Department of Geriatrics, The First Affiliated Hospital of Hunan University of Chinese Medicine, Changsha, China

**Keywords:** artificial intelligence, metabolic disease, intervention, diagnosis, bibliometric analysis

## Abstract

**Background:**

In recent years, the global prevalence of metabolic diseases has continued to rise, emerging as a major public health concern worldwide. Artificial intelligence (AI), with its advanced capabilities in data analysis, pattern recognition, and predictive reasoning, has demonstrated remarkable potential in enhancing the diagnosis, management, and prevention of metabolic disorders.

**Methods:**

Literature searches were conducted in the WOSCC and Scopus databases, followed by database merging and deduplication using R scripts, resulting in a final corpus of 1,059 publications for analysis. Bibliometric analysis tools, including VOSviewer and CiteSpace, were subsequently employed to visualize and quantitatively assess the distribution of publications by country and institution, prolific authors, influential journals, citation networks, and emerging research keywords.

**Results:**

Research on AI in metabolic diseases has experienced explosive growth, with publication output increasing by 394% over the past 5 years, paralleling the broader expansion of AI technologies. China, the United States, and the United Kingdom have emerged as the leading contributors in this domain, with China contributing the largest share (21.87%), followed by the United States (17.10%). Among high-output institutions, Institutions from China contributed the most publications (298), while Harvard Medical School in the United States demonstrates the strongest academic influence. Nieuwdorp M stands out as the most prolific and highly cited author, with Kupusinac and Aleksandar also recognized for their significant contributions to the field. Scientific Reports ranks as the most productive journal, whereas Atherosclerosis is identified as one of the most authoritative journals among high-output publications. The co-occurrence of keywords such as “machine learning,” “deep learning,” “data mining,” “metabolomics,” and “diagnosis” reveals the application of artificial intelligence and advanced data processing techniques in metabolic diseases.

**Discussion:**

This study provides a comprehensive bibliometric overview of the evolution and research trends of AI in the diagnosis and intervention of metabolic diseases. The analysis highlights three major research frontiers: AI-assisted prevention using smart devices, multimodal diagnostic approaches, and intervention strategies guided by large language models (LLMs). Overall, the findings offer valuable insights into the ongoing transformation of metabolic disease management through AI technologies and lay the groundwork for future research aimed at advancing intelligent diagnostic and therapeutic systems.

## 1 Introduction

Metabolic diseases refer to a group of chronic, complex, and multifactorial conditions that arise from disturbances in the body’s metabolic processes. In recent years, the global incidence of these diseases has increased steadily, establishing them as a major public health concern worldwide (1). This category includes a wide range of disorders such as obesity, type 2 diabetes (T2D), insulin resistance (IR), non-alcoholic fatty liver disease (NAFLD), cardiovascular diseases, and metabolism-related cancers (2). Owing to the close interconnections among these conditions, patients frequently experience multiple metabolic disorders concurrently (3). These diseases are typically long-term and closely associated with lifestyle factors, including diet, physical activity, and behavioral habits. Unfortunately, prevention and management are often neglected, leading to late diagnosis when the diseases have already progressed to irreversible stages. Therefore, leveraging advanced technologies to support early detection, continuous monitoring, and proactive management of metabolic diseases, particularly obesity, diabetes, and cardiovascular disorders, is of vital importance in reducing their overall incidence and health burden.

Artificial intelligence (AI) technology is advancing at an unprecedented pace and has achieved substantial success in medical applications. From a functional standpoint, AI can be broadly divided into three main categories: information exploration, information learning, and information-based inference. Its core technologies, such as computer vision (CV), machine learning (ML), deep neural networks, and natural language processing (NLP) (4–6), serve as the foundation for these capabilities. These AI-driven methods have significantly enhanced various aspects of healthcare, including medical image interpretation, disease risk prediction, diagnostic decision support, drug discovery, and the design of personalized treatment strategies (7, 8).

Given the high complexity, chronic progression, and strong dependence on lifestyle factors, the precise diagnosis, early intervention, and personalized management of metabolic diseases remain major clinical challenges. Conventional diagnostic and treatment approaches are often limited in their ability to support large-scale population screening, integrate and analyze complex datasets, and enable real-time, dynamic health monitoring (9). In contrast, AI, with its advanced capabilities in data processing, pattern recognition, and predictive modeling, offers transformative potential for overcoming these limitations. In recent years, AI research and applications in the domain of metabolic diseases have expanded rapidly, with primary focuses on disease prediction, early diagnosis, and targeted intervention (10–13).

Bibliometric analysis, as a research method that integrates quantitative assessment with visualization techniques, enables the systematic exploration of relationships, patterns, and developments within the scientific literature. It serves as a valuable tool for researchers to comprehensively understand the current state of research, identify emerging hotspots, and forecast future trends within a specific field (14). Accordingly, to elucidate the intrinsic connections between metabolic diseases and AI, this study conducts a bibliometric analysis of relevant publications spanning the years 2000–2024.

## 2 Materials and methods

### 2.1 Source database and search strategy

To ensure the reliability and comprehensiveness of the research, this study employed an integrated approach combining the Web of Science (WoS) and Scopus databases for bibliometric visualization analysis. Data retrieval was conducted on both WoS and Scopus databases on July 31, 2025. The search strategy for WoS was [TS = (“artificial intelligence” OR “machine learning” OR “deep learning” OR “neural network*” OR “AI”) AND (“metabolic disease*” OR “metabolic syndrome”)] AND [Article type = (article AND reviews)] AND [Time span = (January 2000 to December 2024)]. To ensure consistency, the search strategy used for Scopus was [TITLE-ABS-KEY (“artificial intelligence” OR “machine learning” OR “deep learning” OR “neural network*” OR “AI”) AND TITLE-ABS-KEY (“metabolic disease*” OR “metabolic syndrome”)] AND [DOCTYPE(ar) OR DOCTYPE(re)] AND (PUBYEAR > 1999 AND PUBYEAR < 2025].

**Figure 1:**
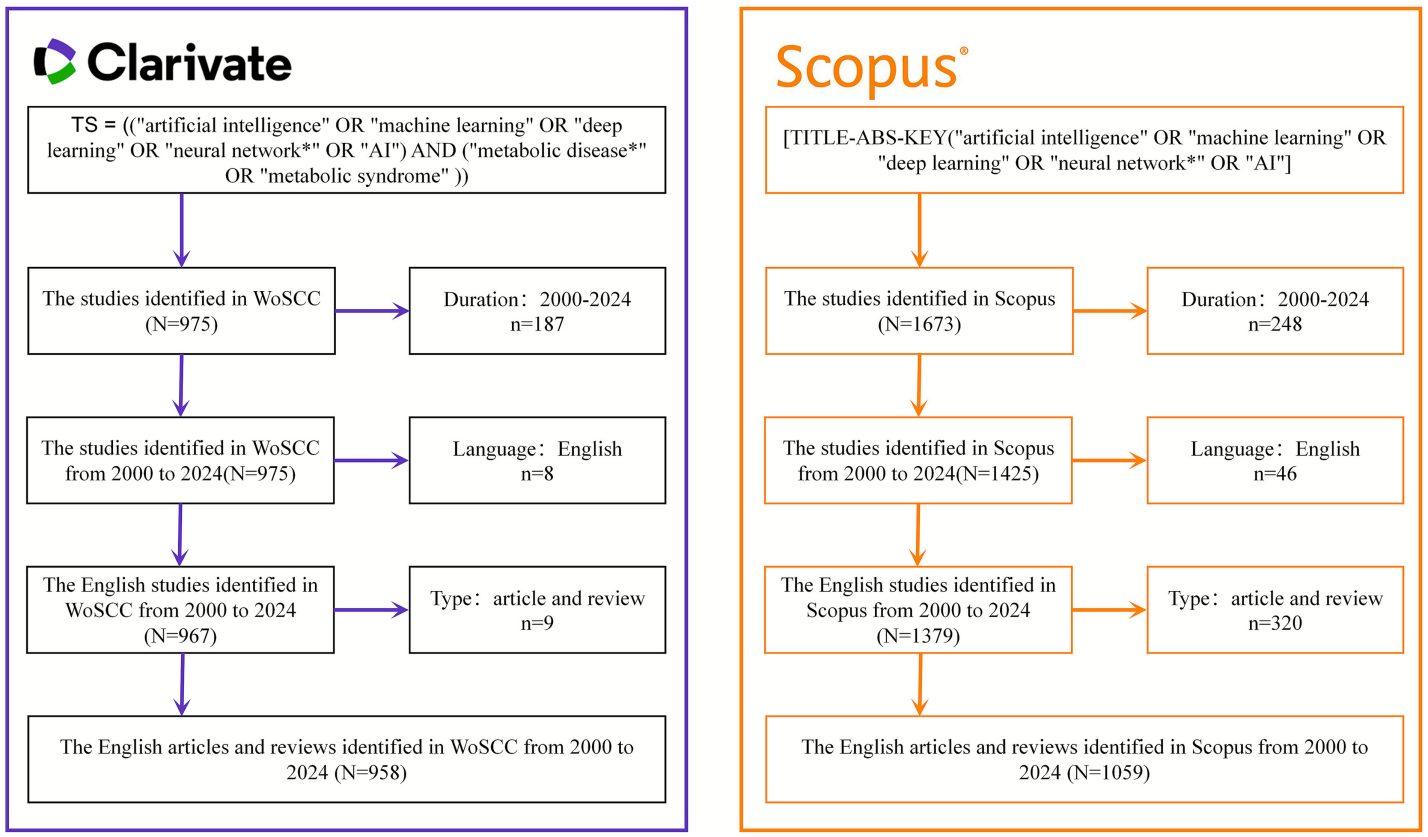
Flowchart of literature inclusion. The figure illustrates the search processes in WoSCC and Scopus, respectively.

Based on the search strategies described above, a total of 958 relevant publications were identified from the WoS database, comprising 853 original research articles and 105 review papers. From the Scopus database, 1,059 relevant publications were retrieved, including 865 research articles and 194 reviews. The detailed process of literature inclusion and exclusion is illustrated in Figure 1.

### 2.2 Database merge

**Figure 2:**
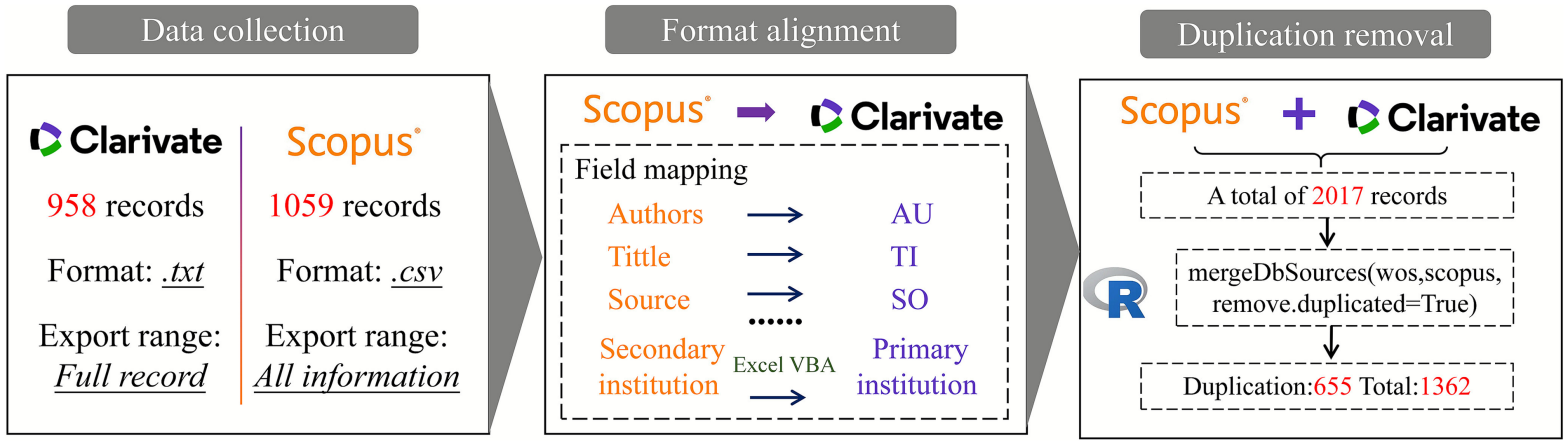
Flowchart of database merging. The figure provides a detailed illustration of the three main components: data collection, format alignment, and duplicate removal of literature.

To further strengthen the reliability of the research, this study integrated data from the WoSCC and Scopus databases using R scripts for deduplication and data harmonization. Initially, all Scopus search results were exported in .csv format, while WoS data were exported in .txt format. Since WoS data offer greater compatibility, its structure was adopted as the baseline, and the Scopus dataset was reformatted accordingly to ensure consistency. Duplicate records were then identified and eliminated through a rigorous multi-step deduplication process. An initial automated match was conducted based on digital object identifiers (DOIs) using a custom R script. For records without DOIs, a fuzzy matching algorithm was applied to compare titles and author names. All potential duplicates flagged by the algorithm underwent manual verification and curation to ensure accuracy. To validate the effectiveness of this process, a random subset of the merged dataset was manually audited. A key challenge in merging the two databases was that institutional affiliations in Scopus were recorded at the secondary level, necessitating a mapping of secondary to primary institutions using Excel VBA code. Additionally, the title fields in the Scopus data were manually adjusted to align with the WoS format. The final step of deduplication and integration was performed in Excel to ensure full consistency across datasets. The complete data merging and deduplication workflow is illustrated in Figure 2.

### 2.3 Data analysis

The integrated dataset from the two databases was analyzed and visualized using a combination of bibliometric tools, including VOSviewer (version 1.6.20), CiteSpace (version 6.4R1), Excel (version 2024), and the R package “Bibliometrix” (version 3.2.1).

VOSviewer is a specialized bibliometric analysis software designed to construct visual knowledge maps from bibliographic data, enabling researchers to extract meaningful insights from extensive bodies of literature. It is widely used for developing collaboration networks, co-citation maps, and co-occurrence visualizations (15). In this study, VOSviewer was applied to conduct co-occurrence analyses across countries, institutions, authors, and keywords to explore global collaboration patterns and thematic relationships.

CiteSpace, another widely used bibliometric tool, offers functions similar to those of VOSviewer but provides additional capabilities that make it particularly valuable for dynamic trend analysis. Specifically, CiteSpace excels in generating burst detection graphs, journal overlay visualizations, and cluster analyses, which allow for the identification of emerging research frontiers and evolving topic structures (16, 17). Therefore, in this study, CiteSpace was utilized to produce burst diagrams, clustering maps, and related visualizations, ensuring a more comprehensive and in-depth interpretation of the bibliometric results.

The Bibliometrix package, an R-based bibliometric analysis tool, offers a comprehensive suite of robust statistical algorithms and high-quality analytical functions (18). In this study, Bibliometrix was employed to perform bibliometric statistical analyses and supplementary evaluations, enhancing the depth and reliability of the overall research findings.

## 3 Results

### 3.1 Bibliometric analysis of annual publications

**Figure 3:**
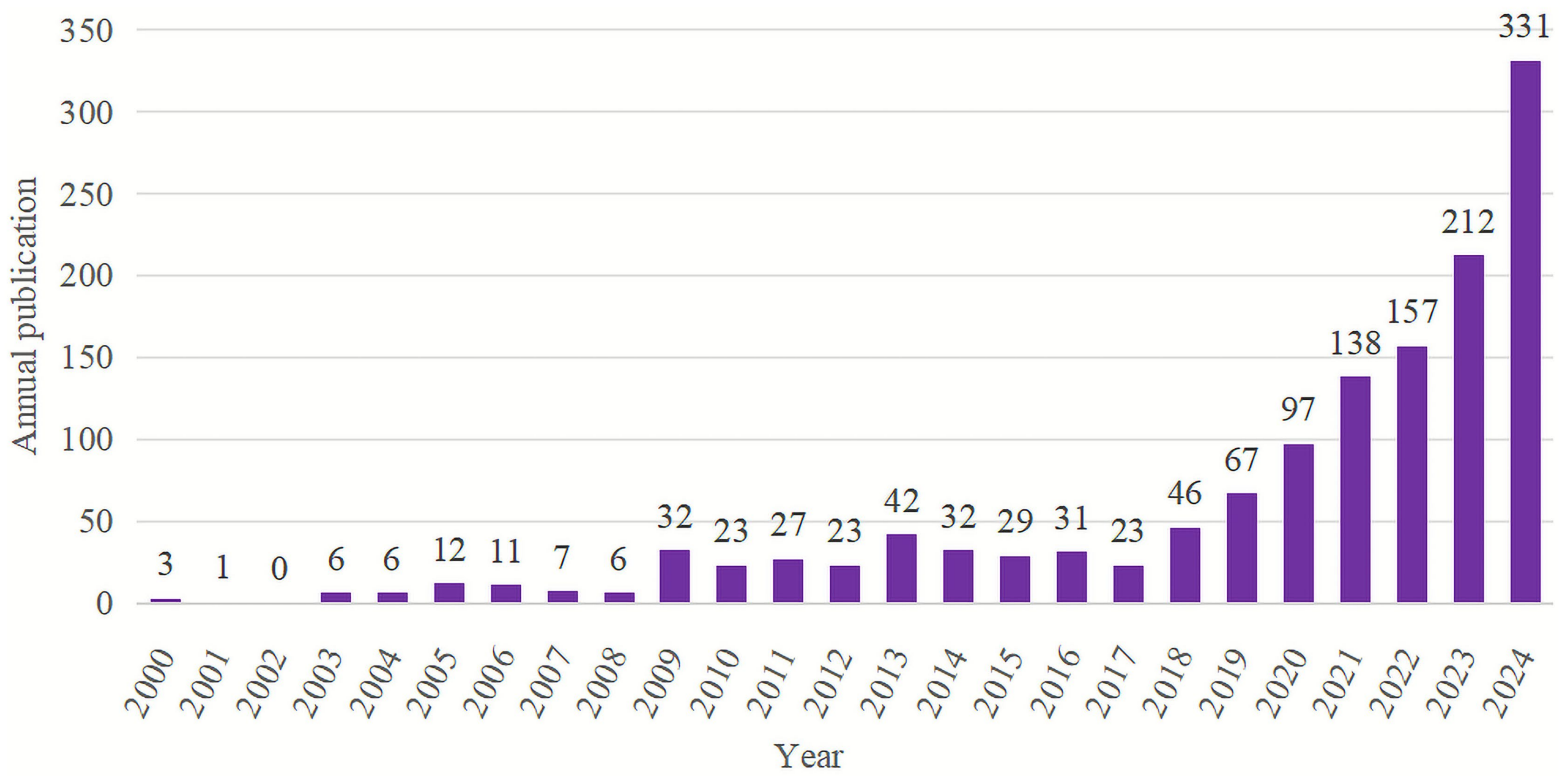
Annual statistical chart of published papers and trend fitting chart. The *X*-axis represents the year, while the *Y*-axis shows the annual publication count in this field.

Using the Bibliometrix package, bibliometric statistical analysis was conducted to assess publication trends from 2000 to 2024, and the resulting data were visualized as a bar chart in Excel (Figure 3). During this period, a total of 1,362 articles related to AI and metabolic diseases were published, averaging 56.75 publications per year. The overall trend reveals a clear stage-wise growth in research output. From 2000 to 2008, publication numbers remained relatively low, reflecting the exploratory stage of AI applications in metabolic disease research. Between 2009 and 2019, publication activity exhibited a gradual and fluctuating rise, marking a period of foundational development. However, from 2020 to 2024, the number of related studies increased sharply, coinciding with the rapid advancement and widespread adoption of AI technologies. This notable upward trend underscores the growing academic and clinical interest in applying AI as an auxiliary tool for the diagnosis, prevention, and treatment of metabolic diseases (see Table 1).

**Table 1:** Content of the search strategy.

Search criteria	Content
WoSCC search strategy	TS = (“artificial intelligence” OR “machine learning” OR “deep learning” OR “neural network*” OR “AI”) AND (“metabolic disease*” OR “metabolic syndrome”)]
Scopus search strategy	TITLE-ABS-KEY (“artificial intelligence” OR “machine learning” OR “deep learning” OR “neural network*” OR “AI”] AND TITLE-ABS-KEY [“metabolic disease*” OR “metabolic syndrome”)]
Time span	January 1, 2000–December 31, 2024
Document type	Article and review
Language	English

### 3.2 Bibliometric analysis of countries and institutions

**Figure 4:**
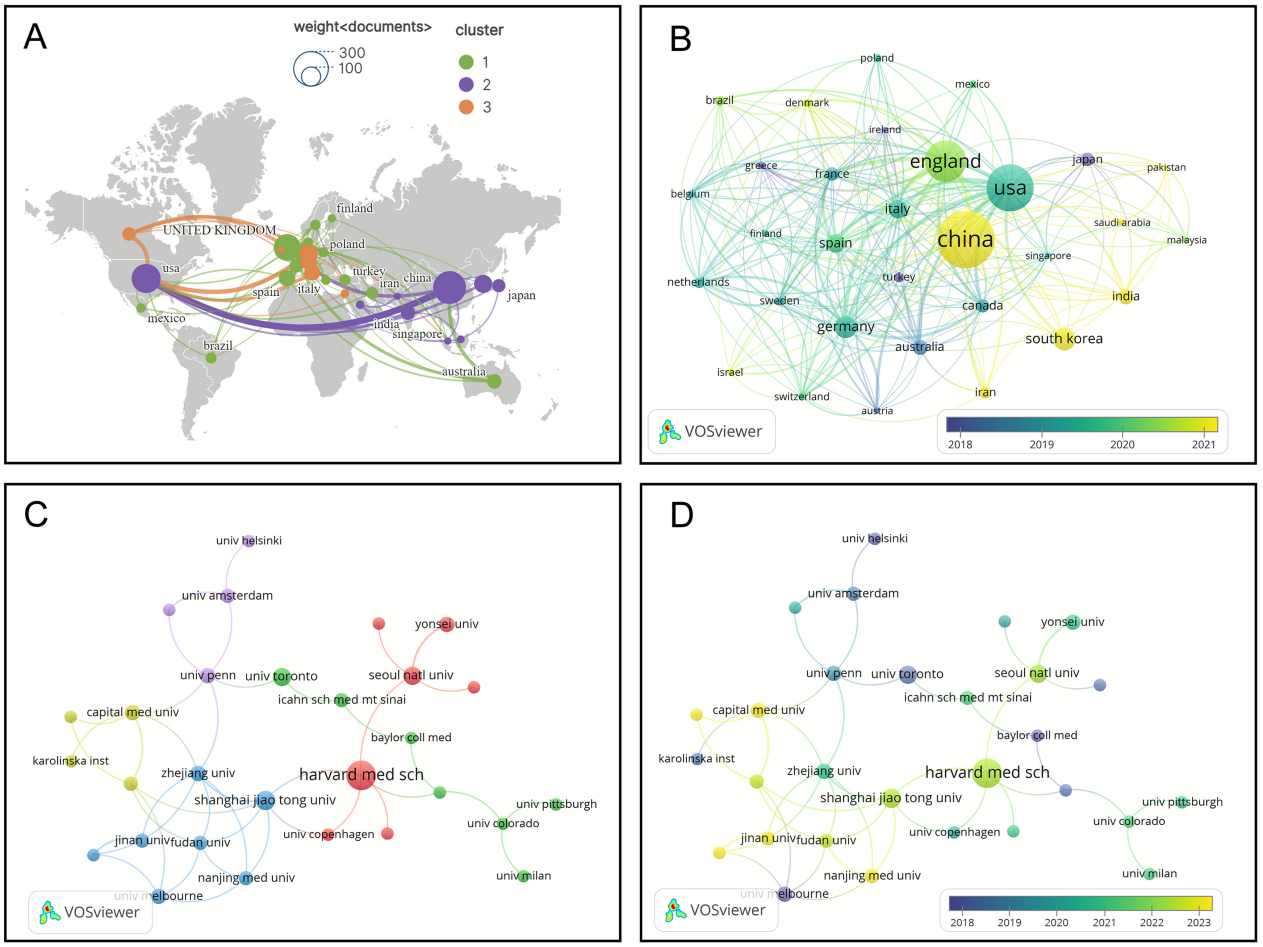
Analysis of countries and institutions’ publications. **(A)** Network diagram of collaborative relations between the top 31 most productive countries, the node size corresponds to publication volume, the color represents cluster affiliation, and both the color saturation and width of the inter-node connections indicate the strength of collaboration between nodes. **(B)** Co-occurrence overlay map of the top 31 most productive countries, and the color brightness of the nodes corresponds to their temporal occurrence, where higher brightness indicates more recently proposed concepts, while lower brightness corresponds to earlier origins. **(C)** Co-occurrence network diagram of the top 29 most productive institutions. **(D)** Co-occurrence overlay map of the top 29 most productive institutions.

Research on AI-assisted treatment of metabolic diseases has been conducted across 124 countries. Among these, China, the United States, and the United Kingdom rank as the top three contributors, accounting for 17%, 13%, and 11% of total publications, respectively, thereby establishing themselves as the leading nations in this research domain (Table 2). As illustrated in Figure 4A, the international collaboration network reveals three major clusters. The first and largest cluster is centered around China and the United States, forming strong cooperative ties with countries in East and Southeast Asia. The second cluster, led by Germany, demonstrates a highly cohesive internal structure with extensive external collaboration, reflecting Germany’s pivotal role in connecting regional and global research efforts. The third cluster revolves around the United Kingdom, featuring dense intra-European cooperation with Germany, France, Spain, and other nations, while also maintaining robust transnational collaborations with both China and the United States. Figure 4B, which presents a time-overlay visualization, highlights the temporal evolution of global research activity. According to this analysis and the data summarized in Table 2, early studies were primarily concentrated in the United States and the United Kingdom, with an average publication year between 2014 and 2016. In contrast, China’s average publication year of 2020 reflects its rapid expansion and increasing influence over the past 5 years, solidifying its position as the leading contributor in the field of AI-assisted research on metabolic diseases.

**Table 2:** Top 10 most productive countries in terms of publications.

Rank	Country	Articles	Articles %	TLS	APY	TC	AC	ACY
1	China	298	21.87	107	2021.66	4,523	15.17	37.25
2	America	233	17.10	190	2019.43	7,557	32.43	19.42
3	England	198	14.53	196	2020.37	7,459	37.67	19.72
4	Germany	92	6.75	140	2019.38	3,879	42.16	7.76
5	Korea	90	6.61	21	2020.91	1,288	14.31	8.98
6	Spain	73	5.36	77	2019.93	1,279	17.52	6.08
7	Italy	72	5.29	68	2019.36	1737	24.12	5.78
8	Australia	58	4.26	75	2018.50	2,399	41.36	4.14
9	India	57	4.19	33	2021.49	974	17.08	7.13
10	Canada	50	3.67	75	2018.92	1,263	25.26	3.57

TLS, total link strength; APY, average publication year, TC, total citation, AC, average citation, and ACY, average citation per year.

A total of 2,005 institutions have contributed to research in this field. Among the top 10 high-output institutions, four are Chinese universities, underscoring China’s increasingly active role in advancing AI-based research on metabolic diseases. Prominent American institutions, such as Harvard University, have also played a central role in shaping this research area. Figures 4C,D illustrate the co-occurrence network of publishing institutions and the corresponding timeline overlay, respectively. As shown in these graphs, active institutions in this domain are primarily grouped into five distinct clusters. Cluster 1 (red) comprises seven nodes, with Seoul National University serving as a key collaborative hub, maintaining strong partnerships with Yonsei University and Harvard Medical School. Cluster 2 (blue) also includes seven nodes, where the Icahn School of Medicine at Mount Sinai and Baylor College of Medicine demonstrate the closest cooperation. Similarly, Cluster 3 (green) encompasses seven institutions, with Fudan University actively collaborating with several members within the cluster and maintaining significant connections with Nanjing Medical University and the University of Melbourne. Cluster 4 (yellow) consists of four institutions, where Capital Medical University, Peking University, and Harbin Medical University engage in close collaboration and exhibit strong research ties with the Karolinska Institute in Sweden. Cluster 5 (purple) includes four institutions, with the University of Amsterdam occupying a central position and maintaining close research relationships with the University of Helsinki, the University of Oxford, and the University of Pennsylvania. Early-stage research (2000–2010) was primarily concentrated in renowned medical centers across Europe and North America, such as Harvard Medical School, the Icahn School of Medicine at Mount Sinai, and the University of Toronto. However, after 2015, Chinese institutions rapidly expanded their contributions, with Shanghai Jiao Tong University, Fudan University, Capital Medical University, and Zhejiang University emerging as key players with notably later average publication years.

### 3.3 Bibliometric analysis of active authors

**Figure 5:**
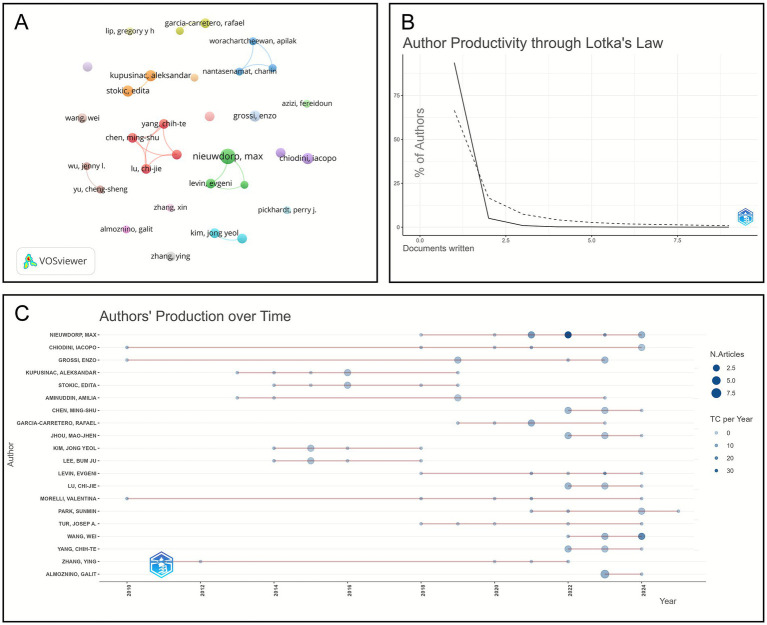
Analysis of authors publications. **(A)** Co-authorship map of the top 31 most productive authors, the node size corresponds to publication volume, the color represents cluster affiliation, and both the color saturation and width of the inter-node connections indicate the strength of collaboration between nodes. **(B)** Author productivity through Lotka’s Law, the solid line represents the theoretical distribution, while the dashed line shows the distribution of actual data. **(C)** Authors’ production over time, the node size corresponds to the author’s annual publication output, while the connection length represents the duration of the author’s research engagement in this field.

A total of 8,604 authors have contributed to research within this field. Figure 5A illustrates the co-occurrence network of authors, revealing that the collaboration structure remains relatively fragmented. Most research groups are small, with the largest cluster comprising only four authors. Numerous researchers have yet to form collaborative connections, represented by isolated nodes or small clusters. The most productive author is Nieuwdorp M, who has published nine papers. Several other authors have produced between five and six publications, forming a high-output subgroup in this domain. Collectively, prolific authors are distributed across Asia, Europe, and the Americas, underscoring the global nature of research in this area (Table 3). Cluster 1 consists of four Taiwanese scholars, Chen M-S, Lu C-J, Jhou M-J, and Yang C-T, who specialize in using ML techniques to assess risks and diagnose metabolic syndrome. Cluster 2 includes European researchers, such as Nieuwdorp M, and primarily focuses on applying AI to study gut microbiota and metabolic disorders. Another small group of Thai scholars concentrates on employing data mining methods to identify metabolic syndrome. The remaining clusters are comparatively small (often comprising only two members) and typically represent collaborations within specific institutions or regions. Figure 5B depicts Lotka’s Law curve of author output, reflecting the publication distribution pattern in this field. The distribution demonstrates a strong positive skew with a pronounced long-tail pattern, where single-authored papers account for 93.7%, signifying that highly productive authors are rare while the majority belong to low-output groups. Figure 5C displays the timeline of author productivity. The trend indicates that Nieuwdorp Max, Chiodini Iacopo, and Grossi Enzo have sustained high publication rates in recent years. Notably, since 2020, Nieuwdorp Max’s number of publications and citations has risen sharply, positioning him as a central figure in this domain. Scholars, such as Kopusinac Aleksandar and Stokic Edita, have become increasingly active since 2015, whereas researchers from the Asia-Pacific region, such as Chen Ming-Shu, Jhou Mao-Jhen, and Lu Chi-Jie, have recently emerged, reflecting a rapid rise in regional research capacity. In contrast, earlier contributors like Morelli Valentina and Park Sunmin have experienced a decline in influence, while emerging scholars such as Yang Chih-Te and Zhang Ying have gradually increased their productivity and impact, thereby expanding the research community and advancing knowledge dissemination within this field.

**Table 3:** Top 10 most productive authors.

Rank	Author	Articles	Articles %	TLS	APY	TC	AC	ACY
1	Nieuwdorp, Max	9	0.66	9	2021.66	266	29.55	2.69
2	Chiodini, Iacopo	6	0.44	5	2019.50	131	21.83	1.09
3	Kupusinac, Aleksandar	6	0.44	5	2015.50	91	15.16	0.63
4	Stokic, Edita	6	0.44	5	2016.33	85	14.16	0.69
5	Grossi, Enzo	6	0.44	0	2019.33	76	12.66	1.06
6	Aminuddin, Amilia	5	0.37	0	2017.60	39	7.80	0.68
7	Chen, Ming-shu	5	0.37	15	2022.80	27	5.40	2.27
8	Garcia-carretero, Rafael	5	0.37	4	2020.80	70	14.00	1.19
9	jhou, mao-jhen	5	0.37	15	2022.80	27	5.40	2.27
10	kim, jong yeol	5	0.37	5	2015.60	88	17.60	0.53

TLS, total link strength, APY, average publication year, TC, total citation, AC, average citation, ACY, average citation per year.

### 3.4 Bibliometric analysis of published journals

**Figure 6:**
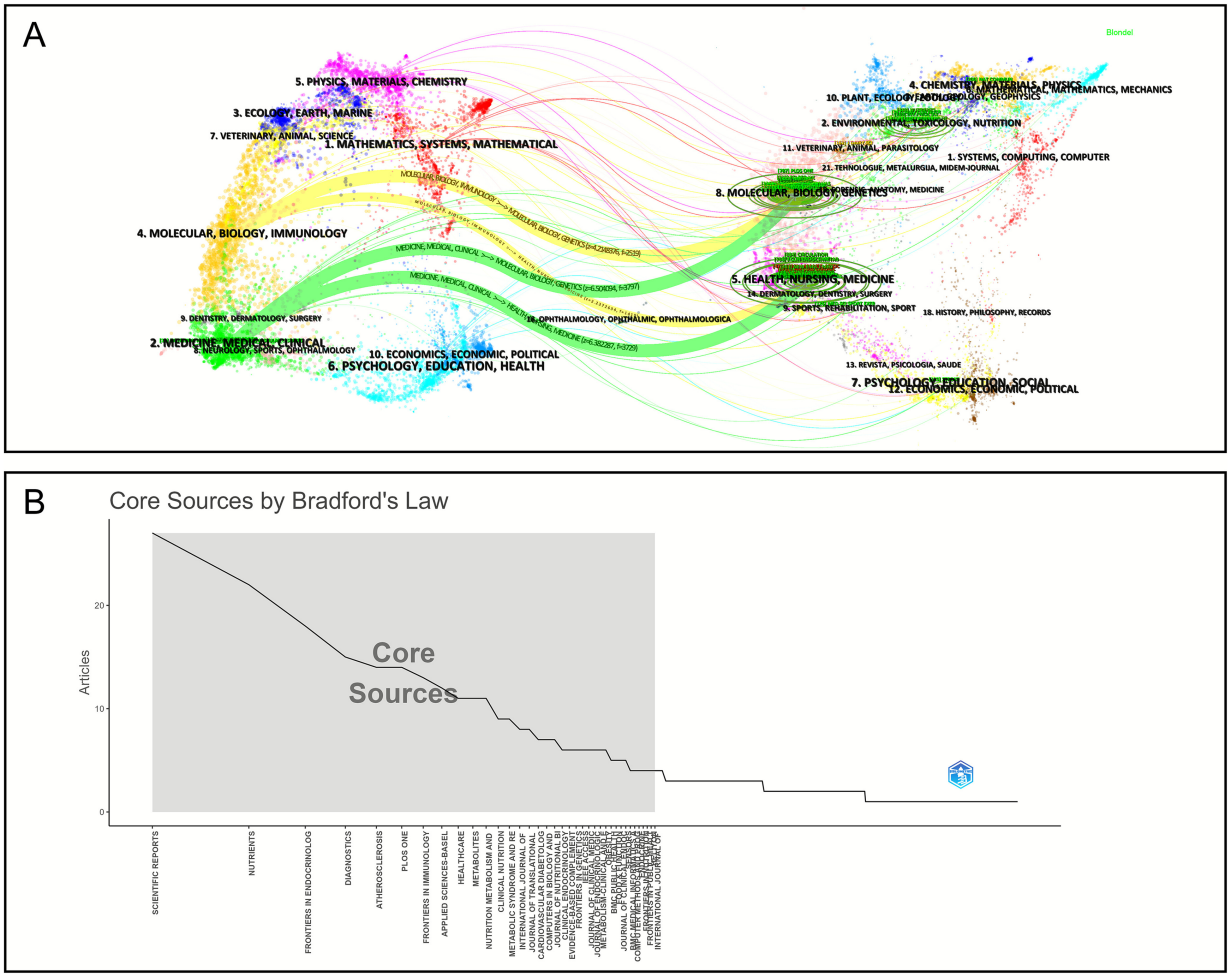
Analysis of source publications. **(A)** Dual-map overlay of journals, where the left side represents the citing journals, the right side shows the cited journals, and the connecting lines in the middle indicate citation relationships between topics. **(B)** Bradford’s Law core journal screening and ranking chart, the journals located within the gray area represent the core journals in this field.

Figure 6A illustrates the dual-map overlay of journals within this field, providing insight into the citation relationships between citing and cited journals and thereby revealing the pathways of interdisciplinary knowledge exchange. The trajectories displayed in the figure indicate that the knowledge flow in this research domain primarily comprises the following components: the yellow trajectory represents the transfer of knowledge from the “molecular biology, genetics” field to the “molecular biology, immunology” field (*z* = 4.21, *f* = 2,519), as well as from the “health, nursing, medicine” field to the “molecular biology, immunology” field (*z* = 4.21, f = 2,519). Meanwhile, the blue trajectory signifies the flow from “molecular biology, genetics” to the “medicine, medical, clinical” field (*z* = 6.50, *f* = 3,797) and from “health, nursing, medicine” to the “medicine, medical, clinical” field (*z* = 6.38, f = 3,729).

Figure 6B presents the core journal distribution map derived from Bradford’s Law. The figure reveals that core journals in this field are densely clustered, accompanied by a substantial number of peripheral journals, a typical feature of interdisciplinary research. Table 4 lists the top 10 journals ranked by publication volume in this area. Collectively, these journals have published 191 papers, representing 14.02% of the entire dataset. Diagnostics exhibit the highest total link strength (TLS = 20), followed by Scientific Reports (TLS = 12), Nutrients (TLS = 10), and PLOS One (TLS = 10), signifying their strong centrality within the literature co-occurrence network and their pivotal role in disseminating knowledge across the field. Regarding publication output and citation performance, Scientific Reports leads as the top comprehensive journal (Articles = 32, TC = 466), with an average publication year of 2020, underscoring its sustained cumulative advantage. Atherosclerosis records the highest average citation count (AC = 31.93, APY = 2012.00), reflecting its concentration of high-impact studies. In contrast, Frontiers in Endocrinology, Nutrients, and Metabolites (APY ≈ 2022) have been particularly active in recent years, maintaining average citation counts between 10 and 12, indicative of their emerging growth and increasing influence in the field.

**Table 4:** Top 10 most productive journals.

Rank	Source	Articles	TLS	APY	AC	IF^2022^	JCR
1	Scientific reports	32	12	2021.84	14.56	3.9	Q1
2	Frontiers in Endocrinology	25	8	2022.96	12.36	4.6	Q1
3	Nutrients	25	10	2022.16	10.68	5.0	Q1
4	Metabolites	21	1	2022.52	10.23	3.7	Q2
5	Plos One	21	10	2020.38	27.95	2.6	Q2
6	Diagnostics	16	20	2022.50	7.75	3.3	Q1
7	Atherosclerosis	15	1	2012.00	31.93	5.7	Q1
8	Frontiers in immunology	13	1	2022.38	27.61	5.9	Q1
9	Applied sciences-basel	12	9	2021.16	10.66	2.5	Q2
10	Healthcare	11	4	2022.27	10.09	2.7	Q2

TLS, total link strength, APY, average publication year, AC; average citation.

### 3.5 References analysis

**Figure 7:**
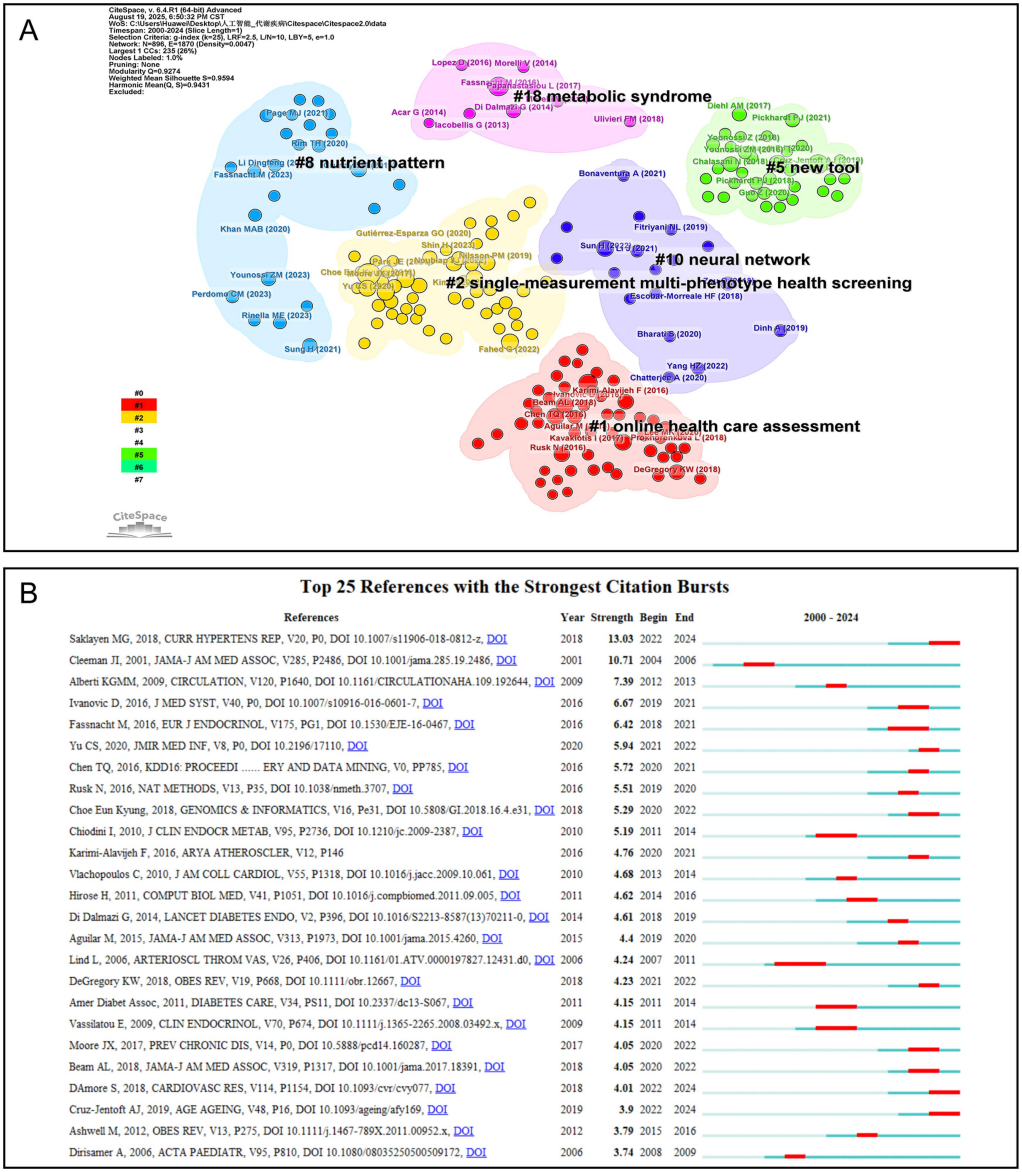
Reference analysis. **(A)** References clustering graph, the figure displays the clustering results of relevant references using the mutual information method, where the black text indicates the theme of each cluster. **(B)** Top 25 references with the strongest citation bursts, the left section displays reference information, while the right area shows the temporal burst of attention each reference received.

Figure 7A illustrates the reference clustering analysis of the merged dataset generated by CiteSpace. The visualization comprises 896 nodes and 1,870 links, with a modularity value (Q) of 0.9274 and a harmonic mean (Q, S) of 0.9431, indicating strong clustering reliability and structural significance. Based on mutual information, six primary clusters were identified: online health care assessment (Cluster #1), single-measurement multi-phenotype health screening (Cluster #2), new tool (Cluster #5), nutrient pattern (Cluster #8), neural network (Cluster #10), and metabolic syndrome (Cluster #18). Each cluster demonstrates a close association with the overarching research theme. Figure 7B presents the burst detection analysis of influential references in this field. The most prominent citation burst is associated with the article by Saklayen (23), which focuses on the epidemiology and risk factors of metabolic syndrome (Strength = 13.0263, Burst time: 2022–2024). This work has been extensively cited in later studies to establish baseline risks and define disease spectrum boundaries across populations. The earliest burst was observed in the paper by Cleeman JI (2001), which introduced the ATP III guidelines (strength = 10.7144, burst time: 2004–2006). This publication exerted a profound influence on clinical lipid management and the diagnostic criteria for metabolic syndrome. The longest-lasting burst was detected in the article by Lind L (2006), addressing vascular and atherosclerosis phenotypes (strength = 4.2374, burst time: 2007–2011).

### 3.6 Keyword analysis

#### 3.6.1 Analysis of keyword co-occurrence

**Figure 8:**
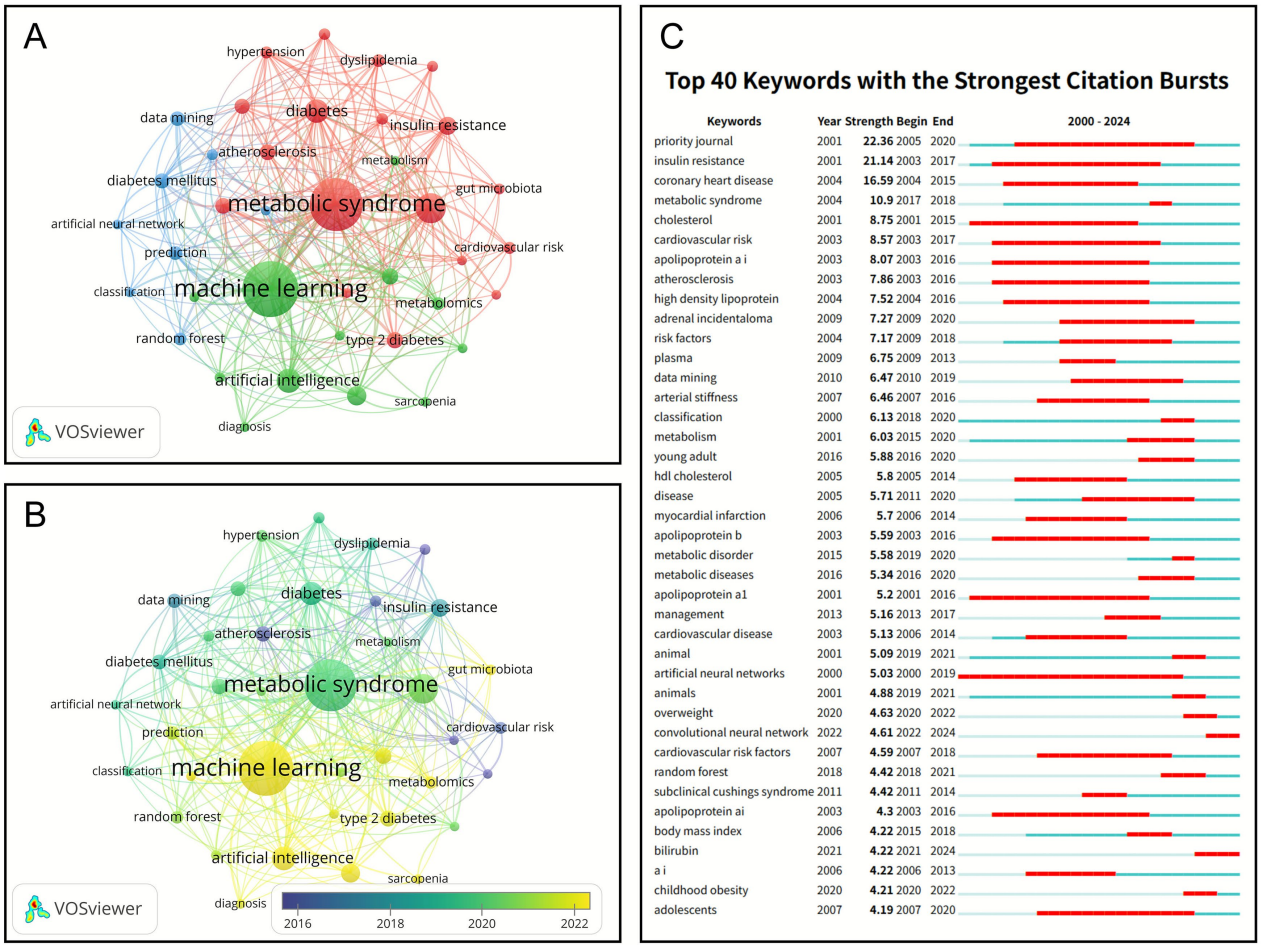
Keyword analysis. **(A)** Co-occurrence network diagram of the top 38 most frequent keywords, the node size corresponds to publication volume, the color represents cluster affiliation, and both the color saturation and width of the inter-node connections indicate the strength of collaboration between nodes. **(B)** Co-occurrence overlay map of the top 38 most frequent keywords; the color brightness of the nodes corresponds to their temporal occurrence, where higher brightness indicates more recently proposed concepts, while lower brightness corresponds to earlier origins. **(C)** Top 25 keywords with the strongest citation bursts, the left section displays reference information, while the right area shows the temporal burst of attention each reference received.

This study identified a total of 3,352 author keywords, among which 38 appeared more than 13 times and were therefore classified as high-frequency terms for the co-occurrence analysis shown in Figure 8A. The figure reveals three distinct clusters. Cluster 1 (red) comprises 18 high-frequency keywords, with “metabolic syndrome,” “obesity,” “diabetes,” and “IR” being the most prevalent. This cluster primarily reflects research centered on metabolic syndrome and related metabolic disorders. Other closely associated keywords within this cluster, such as “T2D,” “atherosclerosis,” “inflammation,” and “hypertension,” suggest that these conditions frequently co-occur and are interconnected in metabolic disease studies. Cluster 2 (green) is organized around the core term “ML” and includes both technical concepts, such as “AI” and “deep learning,” as well as biomedical terms like “biomarkers” and “metabolomics.” This cluster emphasizes the growing integration of AI-driven approaches in the diagnosis and management of metabolic diseases. Cluster 3 (blue) contains eight nodes, featuring keywords such as “data mining,” “prediction,” and “random forest,” which are closely associated with “diabetes mellitus,” indicating a research focus on disease prediction and classification through data mining and ML techniques. Figure 8B presents an overlay visualization with a timeline, illustrating that the keywords in Cluster 2 appear in lighter shades, signifying their recent emergence as contemporary research topics, while earlier, darker-colored keywords, such as “metabolic syndrome” and “cardiovascular disease risk,” reflect the longer-standing research foundation of metabolic disorders.

#### 3.6.2 Analysis of keywords citation burst

In bibliometric visualization analysis, sudden increases in keyword frequency can reveal emerging trends and the evolution of research topics. A significant rise in a keyword’s frequency over a specific period is referred to as a “burst,” which typically signifies that the topic has become a research hotspot during that timeframe. Figure 8C displays the keyword burst diagram for this study. Among the early burst keywords, terms such as “Cholesterol” and “Apolipoprotein A1” began exhibiting bursts from 2001, persisting until 2015 or 2016. These terms relate to lipid metabolism and atherosclerosis, indicating that initial research primarily focused on cardiovascular disease risk factors. Simultaneously, keywords including “IR,” “atherosclerosis,” and “cardiovascular risk” also experienced notable bursts in the early 2000s. In this analysis, the top three keywords with the highest burst intensities are “priority journal” (22.36), “IR” (21.14), and “Coronary Heart Disease” (16.59). Notably, “IR,” as a central concept in metabolic disease research, exhibits both the longest burst duration and the highest burst intensity, highlighting its sustained prominence in the field.

## 4 Discussion

### 4.1 General information

This study employs visualization and bibliometric analysis to map the research landscape of AI-assisted diagnosis and treatment of metabolic diseases. We collected data on annual publication counts, contributing countries, institutions, journals, authors, references, and keywords to identify research hotspots and track the field’s developmental trajectory. To enhance the comprehensiveness of the dataset, journal searches were conducted in both the WOSCC and Scopus databases, followed by a programming-based method to merge the two sources efficiently. A total of 958 English-language articles were retrieved from WOSCC and 1,059 from Scopus; after removing 655 duplicates, the final dataset comprised 1,362 articles. Statistical analysis of annual publication volumes (Figure 3) revealed a marked surge in publications in recent years. This rapid growth is primarily attributable to several factors: (1) the rising global prevalence of metabolic diseases, which imposes a substantial burden on healthcare systems; (2) recent technological advancements in AI, alongside improved computational capabilities, which have facilitated easier implementation of AI technologies; and (3) the ongoing application of mature AI techniques in clinical practice, which has generated extensive clinical datasets to support related research.

Analysis of Figure 4A reveals that the co-occurrence network of countries involved in AI-assisted diagnosis and treatment of metabolic diseases encompasses 124 countries, reflecting the extensive international engagement in this field. The network is predominantly organized into three major clusters. The co-occurrence network is primarily divided into three major clusters. Cluster 1, dominated by European countries, laid the early research foundation; Cluster 2, centered on China and the United States and encompassing other Asian nations, possesses the largest scale and has driven the shift in research focus; meanwhile, Cluster 3, though smaller in volume, demonstrates outstanding influence. Figure 4B illustrates the temporal distribution of research focus, showing that early studies were primarily concentrated in Europe and North America, with a gradual expansion toward Asia and other emerging economies over time. Figure 4C depicts the institutional co-occurrence network, comprising 2,005 institutions worldwide that have contributed to research in this domain. The extensive participation of institutions underscores the breadth of the global research ecosystem, with strong collaborative intensity often observed within regionally distributed clusters. The overlay visualization in Figure 4D indicates that Chinese institutions have been particularly active over the past decade, whereas European and American institutions exhibited higher activity in earlier years, consistent with the trends observed in the country-level co-occurrence network.

This study identified a total of 8,604 authors contributing to AI-assisted diagnosis and treatment of metabolic diseases. The global author collaboration network, depicted in Figure 5A, appears dispersed, reflecting a relatively fragmented pattern of collaboration. The Lotka’s Law curve in Figure 5B demonstrates that publication output in this field is highly skewed, with high-output authors being rare and the majority of publications concentrated among low-output groups, and this pattern adequately explains the phenomenon of dispersed research clusters. As shown in Figure 5C and Table 3, Nieuwdorp Max emerges as both a high-output author and the individual with the highest average citation count per paper, highlighting his substantial contributions to the field, particularly in endocrinology and metabolic diseases (19, 20). Chiodini Iacopo ranks second in terms of publication and citation metrics, maintaining a sustained research focus on dietetics and endocrinology (19). Kupusinac Aleksandar conducted early research on the application of AI technologies to support the treatment of cardiovascular metabolic diseases, thereby laying a foundational basis for subsequent studies in the field (21). Additionally, Kupusinac Aleksandar and Stokic Edita have collaborated, co-authoring an early study employing ML-based clustering analysis to examine the relationship between metabolic syndrome and nutrient intake (22).

The dual-overlay citation relationship map in Figure 6A illustrates that knowledge transfer within the medical field remains the central focus, with AI methodologies functioning as supportive tools across various clinical applications. This pattern suggests that advanced computational algorithms are increasingly integrated into clinical research on metabolic diseases, enhancing the precision of diagnostic and therapeutic decision-making. Among the top 10 journals by publication volume, seven are medicine-focused, while three are general journals (Table 4). Figure 6B displays the core journal distribution map based on Bradford’s Law, revealing a dense concentration of core journals accompanied by numerous peripheral journals, which reflects the interdisciplinary nature of the field. Within this distribution, Atherosclerosis emerges as a particularly influential journal, with an impact factor (IF) of 5.7, an average citation count (TC) of 31.93, and a non-open-access status, underscoring its significance in disseminating high-impact research in the domain.

In the reference clustering analysis depicted in Figure 7A, references were grouped based on mutual information, resulting in six distinct clusters, each closely aligned with the themes addressed in this study. Among these, the “online health care assessment” cluster emphasizes the critical role of telemedicine and real-time health monitoring in personalized health management, while the “neural network” cluster highlights the potential applications of AI techniques, including deep learning, in disease diagnosis and clinical decision-making. Figure 7B presents the burst detection analysis of the references, illustrating both the temporal occurrence and intensity of citation bursts. The article with the highest burst intensity is a 2018 review by Saklayen (23), which examines the global epidemiology of metabolic syndrome. This work provides a comprehensive discussion of the definition, underlying mechanisms, and prevention strategies for metabolic syndrome, establishing a foundational reference for subsequent research in the field.

Although research on the application of AI in the diagnosis and treatment of metabolic diseases is not yet as mature as in other medical domains, the significant increase in related publications over the past decade, along with the expanding diversity of research topics, indicates that this field has emerged as a dynamic and promising frontier. AI technologies are increasingly being incorporated into precision medicine, multimodal data analysis, and personalized interventions, highlighting the translational and clinical significance of this interdisciplinary area.

### 4.2 Hotspot directions

In this study, three primary keyword clusters were identified. Cluster 1 predominantly addresses metabolic diseases, including T2D, obesity, and cardiovascular disorders, whereas Clusters 2 and 3 are mainly focused on AI technologies (Figure 8A). Metabolic diseases, as a representative category of complex chronic conditions, exhibit high heterogeneity, with their pathogenesis and progression influenced by multidimensional factors such as genetic predisposition, lifestyle, and environmental exposures (24). Traditional diagnostic and therapeutic approaches often fall short in meeting the demands for individualized, dynamic, and precise health management. In contrast, AI technologies, with their ability to process high-dimensional and multimodal data, offer essential technical support for shifting from a “population-based” to an “individual-specific” medical paradigm (25, 26) In recent years, the integration of AI into metabolic disease research has progressively deepened, with research hotspots systematically evolving toward three key directions: precise prediction, precise diagnosis, and precise intervention.

#### 4.2.1 Early and precise prediction of metabolic disease risk based on AI and data mining technologies

The cluster encompassing keywords such as “prediction,” “data mining,” and “random forest” (Cluster #3) represents a well-defined research domain centered on data-driven disease prediction and risk assessment, reflecting a systematic research direction within the field. This is supported by the co-occurrence network analysis (Figure 8A), which demonstrates strong internal linkages among these terms, highlighting their central role in the current research landscape. In recent years, the prevalence of metabolic diseases, including T2D, NAFLD, and cardiovascular disorders, has steadily increased, creating a growing need for early risk assessment and preventive strategies. ML approaches, particularly interpretable models such as random forests and XGBoost, have been widely applied to predict metabolic diseases (27–29). The proliferation of advanced intelligent devices and sophisticated data-processing techniques has further facilitated the integration of AI into daily health monitoring, offering new opportunities for precise disease prediction. Smart wearable devices, such as continuous glucose monitoring systems and wristbands, can capture various physiological signals in real time, including blood glucose, heart rate, and physical activity. By applying time-series algorithms to extract dynamic patterns, these devices enable early detection of disease risks and support timely health interventions (13, 30, 31). Moreover, increasing attention to medical data privacy has driven the development of federated learning (FL) frameworks, which allow collaborative training of deep learning models across distributed datasets without sharing raw patient data, thereby addressing critical privacy concerns (32). FL-based approaches have been extensively validated in metabolic disease research, particularly for predicting T2D and cardiovascular conditions, as evidenced by numerous studies (33, 34). However, there is still a lack of long-term studies evaluating their real-world clinical impact on patient outcomes and cost-effectiveness. Furthermore, the performance of these models across diverse clinical settings requires further extensive validation.

#### 4.2.2 Clinical precision diagnosis of metabolic diseases assisted by neural network technology

The cluster centered on keywords such as “ML,” “deep learning,” “metabolomics,” and “diagnosis” (Cluster #2) highlights the application of AI technologies in the clinical diagnosis of metabolic diseases. In diagnostic workflows, neural networks can perform secondary analyses of test reports, supporting more accurate detection of metabolic disorders. Notably, in medical image-assisted diagnosis, several studies have explored visual-assisted ultrasound techniques for fatty liver disease (35, 36). For cardiovascular disease, intelligent electrocardiogram diagnostic technologies, combined with time-series algorithms, have also established a foundational research basis (37). Traditional neural network paradigms have seen limited application in clinical diagnosis due to constraints in generalizability and robustness (38). To address these limitations, multimodal technologies have emerged as a key direction in intelligent clinical diagnostics. By integrating imaging data from modalities such as B-ultrasound, CT, and MRI with structured clinical data, including laboratory indicators and biochemical parameters, multimodal approaches facilitate high-precision diagnosis of metabolic diseases (39–41). Common implementations include multi-input convolutional neural networks and Transformer-based cross-modal self-attention networks, which establish deep associations between image and text features, enabling joint encoding and analysis of medical images alongside patient records (42, 43). However, most clinical diagnostic models function as “black-box” systems with poor interpretability. Since clinical decision-making requires rigorous analytical justification, a key future challenge is to enhance model performance while ensuring interpretability.

#### 4.2.3 Clinical precision intervention strategies for metabolic diseases based on LLMs

By integrating AI technologies from Cluster #2 and Cluster #3 with the conceptual framework of “online health care assessment” identified in the reference analysis, AI-powered online health management is emerging as a key paradigm for metabolic disease intervention. LLMs, as advanced AI technologies, offer distinct advantages in health management and disease intervention due to their exceptional abilities in knowledge integration and natural language interaction (44). The primary role of LLMs in supporting metabolic disease treatment is to assist clinicians with medical guidance and patient education (45, 46). Long-term self-management is critical for managing metabolic-related conditions, and LLMs can function as virtual coaches to help individuals maintain adherence and self-discipline (47–49). In clinical decision-making, including medical prescriptions, LLMs can provide reference-based support leveraging their broad knowledge base (50, 51). Despite these advantages, ethical and technical challenges remain significant. Safeguarding patient privacy and managing sensitive health data present ongoing difficulties (52), while the hallucination problem inherent in LLMs can compromise reliability (53). In summary, although LLMs hold substantial potential for assisting metabolic disease interventions, their widespread application is currently constrained by regulatory limitations and technical challenges.

### 4.3 Limitations

This study has several limitations. First, although the same raw dataset was utilized, the consistency of results obtained from the three primary bibliometric tools, VOSviewer, CiteSpace, and Bibliometrix, requires further examination. Most of the models discussed in the literature are supported primarily by within-study analyses and lack adequate external validation; consequently, robust confirmation using large, independent (preferably multicenter) clinical cohorts is still required. Since the core objective of this bibliometric study is to leverage quantitative indicators to examine macro-level trends across a large body of literature, it is inherently challenging to perform quality assessments or bias analyses for individual publications.

## 5 Conclusion

This study employs VOSviewer, CiteSpace, and online bibliometric platforms to comprehensively evaluate the current research status, hotspots, and emerging trends in AI-assisted interventions for metabolic diseases. Overall, the majority of publications have appeared in the past decade, with research activity demonstrating consistent year-on-year growth, reflecting the recent rapid expansion of the AI field. Although research output is primarily concentrated in China, the United States, and the United Kingdom, the relatively lower network connectivity and average citation counts suggest that China still has opportunities to strengthen international collaboration. Despite the large number of authors contributing to this domain, high-output authors remain a minority. The field represents an intersection of emerging AI technologies and traditional medical disciplines; while a foundational research base exists, it remains in an exploratory stage due to limitations in technological maturity and interdisciplinary integration. The development of AI for targeted clinical applications will be critical moving forward. Overall, this bibliometric analysis provides a comprehensive overview of the field and offers valuable guidance for future research directions.

## Data Availability

The original contributions presented in the study are included in the article/Supplementary material, further inquiries can be directed to the corresponding authors.
