# Supplementary material for: Trends in AI-based diagnosis and intervention of metabolic diseases: a bibliometric analysis of the literature from 2000 to 2024
Source: Front Med (Lausanne). 2025 Dec 5;12:1698366. doi: 10.3389/fmed.2025.1698366 (PMC12714997; doi:10.3389/fmed.2025.1698366)
Supplement: Supplementary file 1 [file Table_1.DOCX]

Supplementary Material

# Supplementary Tables

Table1 Supplementary Data for National Co-occurrence Analysis

| id | label | cluster | Weight  <Links> | weight<Total link strength> | Weight  <Documents> | Weight  <Citations> | Weigh  t<Norm. citations> | score<Avg. pub. year> | score<Avg. citations> | score<Avg. norm. citations> |
| --- | --- | --- | --- | --- | --- | --- | --- | --- | --- | --- |
| 6 | australia | 1 | 21 | 75 | 58 | 2399 | 81.2841 | 2018.5 | 41.3621 | 1.4014 |
| 7 | austria | 3 | 16 | 36 | 19 | 405 | 20.6824 | 2018.4211 | 21.3158 | 1.0885 |
| 10 | belgium | 1 | 18 | 50 | 23 | 875 | 32.2462 | 2019.1739 | 38.0435 | 1.402 |
| 13 | brazil | 1 | 14 | 24 | 33 | 612 | 29.0027 | 2020.3939 | 18.5455 | 0.8789 |
| 16 | canada | 3 | 18 | 75 | 50 | 1263 | 67.2182 | 2018.92 | 25.26 | 1.3444 |
| 20 | china | 2 | 26 | 107 | 298 | 4523 | 284.4506 | 2021.6611 | 15.1779 | 0.9545 |
| 29 | denmark | 1 | 15 | 43 | 27 | 868 | 33.877 | 2020.6296 | 32.1481 | 1.2547 |
| 37 | england | 1 | 28 | 196 | 198 | 7459 | 312.505 | 2020.3737 | 37.6717 | 1.5783 |
| 41 | finland | 1 | 16 | 26 | 19 | 330 | 21.3416 | 2019.3684 | 17.3684 | 1.1232 |
| 42 | france | 1 | 21 | 66 | 49 | 1996 | 65.9011 | 2018.8776 | 40.7347 | 1.3449 |
| 43 | germany | 3 | 26 | 140 | 92 | 3879 | 155.6076 | 2019.3804 | 42.163 | 1.6914 |
| 45 | greece | 1 | 13 | 25 | 25 | 541 | 23.9743 | 2017.12 | 21.64 | 0.959 |
| 50 | india | 2 | 14 | 33 | 57 | 974 | 64.7355 | 2021.4912 | 17.0877 | 1.1357 |
| 52 | iran | 1 | 9 | 22 | 40 | 488 | 38.3314 | 2021 | 12.2 | 0.9583 |
| 54 | ireland | 3 | 14 | 27 | 13 | 629 | 21.3433 | 2018.0769 | 48.3846 | 1.6418 |
| 55 | israel | 3 | 10 | 15 | 21 | 2040 | 33.2258 | 2020.619 | 97.1429 | 1.5822 |
| 56 | italy | 3 | 22 | 68 | 72 | 1737 | 74.1705 | 2019.3611 | 24.125 | 1.0301 |
| 57 | japan | 2 | 11 | 20 | 48 | 595 | 22.507 | 2017.7292 | 12.3958 | 0.4689 |
| 69 | malaysia | 2 | 8 | 17 | 17 | 508 | 23.0687 | 2020.2353 | 29.8824 | 1.357 |
| 71 | mexico | 1 | 7 | 19 | 25 | 322 | 12.618 | 2019.88 | 12.88 | 0.5047 |
| 74 | netherlands | 1 | 17 | 65 | 37 | 1482 | 53.9729 | 2019.2162 | 40.0541 | 1.4587 |
| 81 | pakistan | 2 | 10 | 18 | 14 | 220 | 13.6241 | 2021.0714 | 15.7143 | 0.9732 |
| 87 | poland | 1 | 11 | 20 | 25 | 369 | 13.7101 | 2019.56 | 14.76 | 0.5484 |
| 93 | saudi arabia | 2 | 9 | 22 | 20 | 517 | 29.1428 | 2022.5 | 25.85 | 1.4571 |
| 96 | singapore | 2 | 15 | 31 | 15 | 380 | 18.5371 | 2019.3333 | 25.3333 | 1.2358 |
| 100 | south korea | 2 | 8 | 21 | 90 | 1288 | 69.9639 | 2020.9111 | 14.3111 | 0.7774 |
| 101 | spain | 1 | 22 | 77 | 73 | 1279 | 61.0168 | 2019.9315 | 17.5205 | 0.8358 |
| 104 | sweden | 1 | 20 | 59 | 30 | 560 | 35.5362 | 2019.0333 | 18.6667 | 1.1845 |
| 105 | switzerland | 3 | 18 | 49 | 25 | 1131 | 56.4317 | 2019.88 | 45.24 | 2.2573 |
| 110 | turkey | 1 | 8 | 10 | 34 | 468 | 20.4571 | 2017.4706 | 13.7647 | 0.6017 |
| 117 | usa | 2 | 29 | 190 | 233 | 7557 | 296.7445 | 2019.4335 | 32.4335 | 1.2736 |

## Table2 Supplementary Data for Institutional Co-occurrence Analysis

| id | label | cluster | Weight  <Links> | Weight  <Total link strength> | Weight  <Documents> | Weight  <Citations> | Weight  <Norm. citations> | Score  <Avg. pub. year> | score<Avg. citations> | Score  <Avg. norm. citations> |
| --- | --- | --- | --- | --- | --- | --- | --- | --- | --- | --- |
| 185 | baylor coll med | 2 | 2 | 2 | 7 | 237 | 6.7419 | 2016.5714 | 33.8571 | 0.9631 |
| 284 | capital med univ | 4 | 5 | 6 | 9 | 109 | 14.8026 | 2023.1111 | 12.1111 | 1.6447 |
| 730 | fudan univ | 3 | 5 | 5 | 8 | 137 | 7.7968 | 2022.5 | 17.125 | 0.9746 |
| 866 | harbin med univ | 4 | 2 | 2 | 7 | 61 | 6.8545 | 2023.1429 | 8.7143 | 0.9792 |
| 873 | harvard med sch | 1 | 5 | 5 | 21 | 335 | 26.3528 | 2022.2381 | 15.9524 | 1.2549 |
| 1039 | icahn sch med mt sinai | 2 | 2 | 2 | 8 | 422 | 21.4673 | 2021.25 | 52.75 | 2.6834 |
| 1280 | jinan univ | 3 | 2 | 3 | 8 | 69 | 7.1408 | 2022.875 | 8.625 | 0.8926 |
| 1328 | karolinska inst | 4 | 1 | 1 | 7 | 171 | 10.1301 | 2018.5714 | 24.4286 | 1.4472 |
| 1393 | korea univ | 1 | 1 | 1 | 7 | 230 | 8.3759 | 2018.4286 | 32.8571 | 1.1966 |
| 1418 | kyung hee univ | 1 | 1 | 1 | 7 | 79 | 4.4457 | 2019.8571 | 11.2857 | 0.6351 |
| 1665 | nanjing med univ | 3 | 4 | 4 | 8 | 124 | 7.9472 | 2023.125 | 15.5 | 0.9934 |
| 1886 | peking univ | 4 | 5 | 6 | 8 | 96 | 8.1927 | 2022.5 | 12 | 1.0241 |
| 2097 | seoul natl univ | 1 | 4 | 5 | 11 | 219 | 17.3538 | 2022.0909 | 19.9091 | 1.5776 |
| 2125 | shanghai jiao tong univ | 3 | 6 | 7 | 12 | 174 | 10.9614 | 2022.25 | 14.5 | 0.9135 |
| 2244 | sun yat sen univ | 3 | 3 | 4 | 7 | 57 | 6.2536 | 2023.2857 | 8.1429 | 0.8934 |
| 2476 | univ amsterdam | 5 | 3 | 4 | 8 | 835 | 20.5805 | 2018.75 | 104.375 | 2.5726 |
| 2552 | univ colorado | 2 | 3 | 3 | 7 | 61 | 3.9673 | 2021.2857 | 8.7143 | 0.5668 |
| 2560 | univ copenhagen | 1 | 2 | 2 | 7 | 292 | 8.2841 | 2020 | 41.7143 | 1.1834 |
| 2610 | univ helsinki | 5 | 1 | 1 | 7 | 171 | 6.3849 | 2013.8571 | 24.4286 | 0.9121 |
| 2701 | univ melbourne | 3 | 4 | 4 | 9 | 88 | 4.6645 | 2017.8889 | 9.7778 | 0.5183 |
| 2705 | univ milan | 2 | 1 | 1 | 7 | 144 | 5.9479 | 2021.1429 | 20.5714 | 0.8497 |
| 2736 | univ oxford | 5 | 2 | 3 | 7 | 794 | 18.4519 | 2020.1429 | 113.4286 | 2.636 |
| 2748 | univ penn | 5 | 5 | 5 | 9 | 721 | 23.5929 | 2019.2222 | 80.1111 | 2.6214 |
| 2753 | univ pittsburgh | 2 | 1 | 1 | 7 | 98 | 3.9731 | 2020.8571 | 14 | 0.5676 |
| 2819 | univ toronto | 2 | 2 | 2 | 11 | 371 | 22.0846 | 2018.4545 | 33.7273 | 2.0077 |
| 2837 | univ washington | 2 | 3 | 3 | 7 | 177 | 6.6062 | 2018.4286 | 25.2857 | 0.9437 |
| 2840 | univ wisconsin | 1 | 1 | 1 | 7 | 319 | 13.0153 | 2021 | 45.5714 | 1.8593 |
| 3266 | yonsei univ | 1 | 1 | 2 | 9 | 181 | 9.5986 | 2020.7778 | 20.1111 | 1.0665 |
| 3285 | zhejiang univ | 3 | 7 | 8 | 9 | 108 | 8.3388 | 2020.7778 | 12 | 0.9265 |

## Table3 Supplementary Data forAuthors Co-occurrence Analysis

| id | label | cluster | Weight  <Links> | Weight  <Total link strength> | Weight  <Documents> | Weight  <Citations> | Weight  <Norm. citations> | score<Avg. pub. year> | score<Avg. citations> | score<Avg. norm. citations> |
| --- | --- | --- | --- | --- | --- | --- | --- | --- | --- | --- |
| 203 | almoznino, galit | 9 | 0 | 0 | 4 | 27 | 2.5435 | 2023.25 | 6.75 | 0.6359 |
| 241 | aminuddin, amilia | 10 | 0 | 0 | 5 | 39 | 1.4149 | 2017.6 | 7.8 | 0.283 |
| 387 | azizi, fereidoun | 11 | 0 | 0 | 4 | 35 | 1.4781 | 2018.75 | 8.75 | 0.3695 |
| 489 | barquero-perez, oscar | 4 | 1 | 4 | 4 | 67 | 2.1579 | 2020.25 | 16.75 | 0.5395 |
| 1100 | chen, ming-shu | 1 | 3 | 15 | 5 | 27 | 1.6337 | 2022.8 | 5.4 | 0.3267 |
| 1196 | chiodini, iacopo | 5 | 1 | 5 | 6 | 131 | 4.7569 | 2019.5 | 21.8333 | 0.7928 |
| 2180 | garcia-carretero, rafael | 4 | 1 | 4 | 5 | 70 | 2.3664 | 2020.8 | 14 | 0.4733 |
| 2429 | groen, albert k. | 2 | 2 | 7 | 4 | 52 | 2.1101 | 2020.75 | 13 | 0.5275 |
| 2434 | grossi, enzo | 12 | 0 | 0 | 6 | 76 | 2.912 | 2019.3333 | 12.6667 | 0.4853 |
| 3115 | jhou, mao-jhen | 1 | 3 | 15 | 5 | 27 | 1.6337 | 2022.8 | 5.4 | 0.3267 |
| 3487 | kim, jong yeol | 6 | 1 | 5 | 5 | 88 | 2.225 | 2015.6 | 17.6 | 0.445 |
| 3727 | kupusinac, aleksandar | 7 | 1 | 5 | 6 | 91 | 3.1377 | 2015.5 | 15.1667 | 0.5229 |
| 3868 | lee, bum ju | 6 | 1 | 5 | 5 | 88 | 2.225 | 2015.6 | 17.6 | 0.445 |
| 3978 | levin, evgeni | 2 | 2 | 8 | 5 | 88 | 4.9398 | 2021.6 | 17.6 | 0.988 |
| 4221 | lip, gregory y h | 13 | 0 | 0 | 4 | 19 | 1.3467 | 2022.75 | 4.75 | 0.3367 |
| 4393 | lu, chi-jie | 1 | 3 | 15 | 5 | 27 | 1.6337 | 2022.8 | 5.4 | 0.3267 |
| 4982 | morelli, valentina | 5 | 1 | 5 | 5 | 129 | 4.2845 | 2018.6 | 25.8 | 0.8569 |
| 5122 | nantasenamat, chanin | 3 | 2 | 8 | 4 | 97 | 2.9906 | 2013.5 | 24.25 | 0.7477 |
| 5227 | nieuwdorp, max | 2 | 2 | 9 | 9 | 266 | 16.4148 | 2021.6667 | 29.5556 | 1.8239 |
| 5512 | park, sunmin | 14 | 0 | 0 | 5 | 60 | 4.5686 | 2023.2 | 12 | 0.9137 |
| 5639 | pickhardt, perry j. | 15 | 0 | 0 | 4 | 306 | 12.3838 | 2021.75 | 76.5 | 3.0959 |
| 5722 | prachayasittikul, virapong | 3 | 2 | 8 | 4 | 97 | 2.9906 | 2013.5 | 24.25 | 0.7477 |
| 6728 | stokic, edita | 7 | 1 | 5 | 6 | 85 | 2.8079 | 2016.3333 | 14.1667 | 0.468 |
| 7261 | vargas-alarcon, gilberto | 16 | 0 | 0 | 4 | 40 | 1.2568 | 2015.75 | 10 | 0.3142 |
| 7478 | wang, wei | 17 | 0 | 0 | 5 | 71 | 10.72 | 2023.2 | 14.2 | 2.144 |
| 7661 | worachartcheewan, apilak | 3 | 2 | 8 | 4 | 97 | 2.9906 | 2013.5 | 24.25 | 0.7477 |
| 7678 | wu, jenny l. | 8 | 1 | 4 | 4 | 68 | 1.9345 | 2021 | 17 | 0.4836 |
| 7862 | yang, chih-te | 1 | 3 | 15 | 5 | 27 | 1.6337 | 2022.8 | 5.4 | 0.3267 |
| 8018 | yu, cheng-sheng | 8 | 1 | 4 | 4 | 68 | 1.9345 | 2021 | 17 | 0.4836 |
| 8215 | zhang, xin | 18 | 0 | 0 | 4 | 52 | 4.1142 | 2023.75 | 13 | 1.0285 |
| 8229 | zhang, ying | 19 | 0 | 0 | 5 | 90 | 3.0671 | 2017.2 | 18 | 0.6134 |

**Table4 Supplementary Data for Keywords Co-occurrence Analysis**

| id | label | cluster | weight<Links> | weight<Total link strength> | Weight  <Occurrences> | score<Avg. pub. year> | score<Avg. citations> | score<Avg. norm. citations> |
| --- | --- | --- | --- | --- | --- | --- | --- | --- |
| 94 | adrenal incidentaloma | 1 | 8 | 14 | 17 | 2016 | 18.9412 | 0.5226 |
| 284 | arterial stiffness | 1 | 5 | 15 | 14 | 2014.5714 | 20.2857 | 0.7345 |
| 289 | artificial intelligence | 2 | 25 | 75 | 65 | 2022.7846 | 25.0923 | 1.3426 |
| 293 | artificial neural network | 3 | 10 | 18 | 13 | 2019.3077 | 8 | 0.3132 |
| 317 | atherosclerosis | 1 | 16 | 36 | 32 | 2016.1562 | 22 | 0.7469 |
| 425 | biomarkers | 2 | 18 | 50 | 33 | 2021.7576 | 10.9091 | 0.6443 |
| 448 | body composition | 2 | 10 | 21 | 14 | 2020.3571 | 16.8571 | 0.7425 |
| 542 | cardiovascular disease | 1 | 22 | 48 | 32 | 2019.9375 | 29.9688 | 1.1955 |
| 545 | cardiovascular diseases | 1 | 12 | 27 | 18 | 2019.5 | 35.3333 | 2.0437 |
| 555 | cardiovascular risk | 1 | 11 | 21 | 20 | 2016.5 | 23.2 | 0.7826 |
| 669 | classification | 3 | 11 | 18 | 13 | 2019.5385 | 10.3846 | 0.5012 |
| 773 | coronary artery disease | 1 | 14 | 26 | 18 | 2016.3889 | 21.6667 | 0.7056 |
| 835 | data mining | 3 | 10 | 36 | 27 | 2017.4815 | 26.037 | 0.7296 |
| 859 | deep learning | 2 | 15 | 52 | 45 | 2022.3111 | 26.2889 | 1.2008 |
| 882 | diabetes | 1 | 24 | 102 | 64 | 2019.4219 | 25.3594 | 1.0246 |
| 889 | diabetes mellitus | 3 | 22 | 47 | 29 | 2018.8621 | 13.4483 | 0.7258 |
| 911 | diagnosis | 2 | 8 | 16 | 13 | 2021.6923 | 12.5385 | 0.7222 |
| 1023 | dyslipidemia | 1 | 14 | 39 | 22 | 2018.9091 | 20.1818 | 0.8933 |
| 1392 | gut microbiota | 1 | 10 | 20 | 17 | 2022.3529 | 35.1176 | 2.2536 |
| 1563 | hypertension | 1 | 17 | 41 | 20 | 2020.15 | 15.8 | 0.9421 |
| 1635 | inflammation | 1 | 17 | 36 | 29 | 2019.8621 | 32.7586 | 1.3475 |
| 1657 | insulin resistance | 1 | 22 | 63 | 41 | 2017.9024 | 19 | 0.8295 |
| 1769 | leptin | 1 | 12 | 13 | 13 | 2015.7692 | 24 | 0.8106 |
| 1872 | machine learning | 2 | 33 | 299 | 288 | 2021.9479 | 16.5833 | 0.8527 |
| 1972 | metabolic disease | 2 | 12 | 15 | 13 | 2021.6923 | 15.0769 | 0.8023 |
| 1973 | metabolic diseases | 1 | 12 | 17 | 14 | 2020.8571 | 12.2857 | 1.1278 |
| 1998 | metabolic syndrome | 1 | 34 | 258 | 259 | 2019.8456 | 12.4247 | 0.6337 |
| 2011 | metabolism | 2 | 7 | 12 | 14 | 2020.1429 | 44.2857 | 1.4881 |
| 2016 | metabolomics | 2 | 12 | 23 | 21 | 2022.7619 | 12.619 | 0.8538 |
| 2164 | nafld | 2 | 12 | 16 | 15 | 2021.0667 | 29.6667 | 1.8832 |
| 2221 | nhanes | 2 | 8 | 19 | 14 | 2022.5714 | 13.5714 | 1.1739 |
| 2302 | obesity | 1 | 34 | 125 | 99 | 2020.4545 | 24.8788 | 1.1489 |
| 2580 | prediction | 3 | 18 | 43 | 26 | 2021.2692 | 34.4231 | 1.355 |
| 2708 | random forest | 3 | 9 | 24 | 21 | 2020.7619 | 28.8571 | 0.9496 |
| 2805 | risk factors | 3 | 10 | 18 | 17 | 2019.8824 | 81.8824 | 2.6897 |
| 2827 | sarcopenia | 2 | 6 | 13 | 13 | 2022.6923 | 15.9231 | 1.0589 |
| 3200 | type 2 diabetes | 1 | 14 | 38 | 34 | 2021.7059 | 14.0882 | 0.7257 |
| 3202 | type 2 diabetes mellitus | 3 | 8 | 14 | 14 | 2020.4286 | 17.9286 | 0.7418 |
